# Supplementary material for: Movement behaviours and physical, cognitive, and social-emotional development in preschool-aged children: Cross-sectional associations using compositional analyses
Source: PLoS One. 2020 Aug 18;15(8):e0237945. doi: 10.1371/journal.pone.0237945 (PMC7433874; doi:10.1371/journal.pone.0237945)
Supplement: Attachment — Submitted filename: Kuzik_Dissertation Study 2_PLOS One_Response to reviewers.docx [file pone.0237945.s001.docx]

| Editor |  |
| --- | --- |
| E.1 | Please ensure that your manuscript meets PLOS ONE's style requirements, including those for file naming. The PLOS ONE style templates can be found at [https://journals.plos.org/plosone/s/file?id=wjVg/PLOSOne_formatting_sample_main_body.pdf](about:blank) and [https://journals.plos.org/plosone/s/file?id=ba62/PLOSOne_formatting_sample_title_authors_affiliations.pdf](about:blank) |
|  | We have gone through the manuscript to ensure we have met PLOS ONE’s style requirements. Title page has been removed as a heading. |
| E.2 | We note that you have indicated that data from this study are available upon request. PLOS only allows data to be available upon request if there are legal or ethical restrictions on sharing data publicly. For information on unacceptable data access restrictions, please see [http://journals.plos.org/plosone/s/data-availability#loc-unacceptable-data-access-restrictions](about:blank#loc-unacceptable-data-access-restrictions).  In your revised cover letter, please address the following prompts:  a) If there are ethical or legal restrictions on sharing a de-identified data set, please explain them in detail (e.g., data contain potentially identifying or sensitive patient information) and who has imposed them (e.g., an ethics committee). Please also provide contact information for a data access committee, ethics committee, or other institutional body to which data requests may be sent.  b) If there are no restrictions, please upload the minimal anonymized data set necessary to replicate your study findings as either Supporting Information files or to a stable, public repository and provide us with the relevant URLs, DOIs, or accession numbers. Please see [http://www.bmj.com/content/340/bmj.c181.long](about:blank) for guidelines on how to de-identify and prepare clinical data for publication. For a list of acceptable repositories, please see [http://journals.plos.org/plosone/s/data-availability#loc-recommended-repositories](about:blank#loc-recommended-repositories).  We will update your Data Availability statement on your behalf to reflect the information you provide. |
|  | Our ethics board requires the original investigators to file an ethics application for external data access. Thus, if readers were interested in performing a secondary data analysis or a replication of our findings, they would need to contact the corresponding author of this paper and we could then submit an ethics application. |
| E.3 | We note you have included a table to which you do not refer in the text of your manuscript. Please ensure that you refer to Table 7 in your text; if accepted, production will need this reference to link the reader to the Table. |
|  | Table 7 is referenced in the first paragraph of the Discussion section page 24, lines 378:  “*However, associations with social-emotional development were less apparent. A summary of findings are presented in Tables 3 and 7.*” |
| Reviewer 1 (R1) |  |
| General | The aim of this study was to examine the relationships between movement behaviours and indicators of physical, cognitive, and social emotional development in preschool-aged children. I strongly thing that the statistical approach has been perfectly chosen, furtherm need to be ore, it also has a large number of analysed variables. Nevertheless, there are some issues that should be considered and which I will now discuss: |
|  | We thank the reviewer for their time spent reviewing this manuscript, and have addressed their comments below. |
| R1.1 (Methods) | 1. Participants and procedures, page 5, line 88. The sample was recruited through a “Sportball”, a program that aims to teach children fundamental sport skills through play, therefore we have a population bias. In this case, it was selected children and families who do physical activity regularly, moreover, the results showed that children had higher values of MVPA than those considered reference values. |
|  | We have made the following change in the Discussion on page 28 lines 439-443:  “*Lastly, convenience sampling from a physical activity program could have limited our generalizability. In fact, the average minutes/day of MVPA in this sample was 40 minutes higher compared to the national average, which could suggest poor generalizability to the broader population of Canadian preschool aged children*”. |
| R1.2 | 2. Participants and procedures, page 6, line 100. Did the subjects have to complete all the tests to be included in the study? minimum percentage? |
|  | We have made the following change to make this clearer on page 15, lines 304-311:  “*From 131 participants, 26 did not receive accelerometers because they were absent from motor development assessments (n=15) or they did not schedule a time for accelerometers to be dropped off after the motor development assessment (n=11). Further, 10 participants did not meet accelerometer wear time criteria. Thus, a total of 95 participants had usable accelerometer data and were included in the analysis. Aside from the analysis of response inhibition (n=93; n=2 software errors) and all motor skills outcomes (n=93, n=2 children chose not to participate), these 95 participants had data for all outcome variables.*” |
| R1.3 (Measures) | 3. Movement behaviours, page 7, line 112. Did you consider the excessive counts? |
|  | We did not consider excessive counts, or vigorous physical activity. Vigorous physical activity measured with accelerometers can be misclassified as moderate physical activity in what is termed the plateau effect.* So, to protect against this inaccuracy, MVPA is a suitable alternative, since it collapses moderate physical activity with higher intensity counts.  *John, D., Miller, R., Kozey-Keadle, S., Caldwell, G., & Freedson, P. (2012). Biomechanical examination of the ‘plateau phenomenon’ in ActiGraph vertical activity counts. Physiological Measurement, 33(2), 219. |
| R1.4 (Results) | 4. Page 15, line 297. The final sample was 95 participants, which was not very large. Did you calculate the statistical power? |
|  | Power calculations were not conducted a priori due to the novel analyses being conducted. However, to address statistical power we have added the following to the discussion on page 28, lines 436-439:  “*Additionally, the analytical sample was relatively small (n=95) and only powered to detect medium-large effect sizes in models with <3 covariates, and large effect sizes in models with ≥3 covariates (i.e., percent of expected adult height, vocabulary, and prosocial behaviour).*” |
| R1.5 | 5. Page 16. Table 2. The greatest co-dependence was shown between MVPA and stationary time, just as stationary time had a greater co-dependence with LPA against sleep. These values anticipate subsequent results in terms of motor skills, which may not be expected. Particularly, the following results could be highlighted: - LPA was significantly and negatively associated with object and total motor skills. Moreover, locomotor and total motor skills also showed composition full models that included the stationary time as a positive component. - In relation to the substitution models, both in locomotor and total motor skills, to replace LPA for stationary time showed a significant and positive effect; as well as, the opposite replacement was significantly negative. Furthermore, to replace LPA for MVPA showed a significant and positive effect on locomotor skills, but no significant results were found in the replacement of stationary time for LPA. Consequently, these results suggest that LPA could be more detrimental in terms of motor skills than the stationary time. How could this be explained?? I This important question has not been considered in the discussion section. |
|  | We agree with the reviewer that this finding was unexpected. Given the novelty of this finding, we feel that instead of offering speculations it presents an opportunity for future research directions. Specifically, by using measurement tools that are better able to distinguish LPA and sedentary behaviours (e.g., correct classification of standing time, context of sedentary behaviours) in a larger more generalizable sample could confirm or deny this finding with confidence, and potentially speculate on mechanisms for any potential relationships. To illustrate this point, we have added the following to the discussion on page 26, lines 388-393:  “*In contrast, LPA was negatively associated with motor skills in regression models and substitution models that subtracted LPA and added stationary time. Future research is needed with tools that more accurately distinguish between sedentary behaviours and LPA in a larger more generalizable sample to better understand how these parts of the movement behaviour composition impact motor skills.*” |
| Reviewer #2 |  |
| General | This paper explores the 24-hour integrated movement behaviours and their association with health outcomes in pre-school aged children. The authors explore outcomes in the domains of: physical, cognitive and social-emotional development. The authors state that physical development has been the primary focus of previous studies in the field, and a limitation of previous studies is that there is little to no consideration for all behaviours over the 24-hour day. This study, therefore, addresses two gaps by exploring movement behaviours as a complete 24-hour profile as well as exploring outcomes in other domains of development. The methods or rigorous and sound, including the statistical analyses. This paper adds to the growing body of literature around the importance of the 24-hour profile of movement behaviours. My specific comments are below. |
|  | We thank the reviewer for their time spent reviewing this manuscript, and have addressed their comments below. |
| R2.1 (Major) | So that the results are easier to follow, I suggest the results are presented in reference to the outcome domains of Physical development, Cognitive development, Social-emotional development (that is, in the text as well as in the Tables). There are a lot of outcomes and it is hard to follow at times. The authors stated in the introduction that a strength of this paper was that associations between movement behaviours and other non-physical development outcomes were explored, so I think this needs to be reflected in how the results are presented. This is in fact how the Discussion is laid out. |
|  | The developmental domains have been indicated in Tables 4-6, as well the results section has been edited to present our findings for each developmental domain. Please see the Results section on pages 15-23 for full details. |
| R2.2 | The language around the time reallocations is at time confusing. Consider using language that better reflects what was actually done, i.e., reallocation or replacing time. Adding/subtracting is technically correct, but it is more true to say that the time is reallocated. E.g., line 348 of Discussion “For instance, adding 30 minutes of MVPA while subtracting 30 minutes of LPA resulted in higher locomotor and object motor skills by 3.28 and 3.99 units” would be clearer as “replacing/reallocating 30 min of LPA with 30-min of MVPA”. |
|  | We can appreciate the reviewer’s point that the term reallocation may be truer to the analysis. However, we took the approach of using adding or + and subtracting or - to make it easier for the reader to follow the results. To better clarify our operational definition representing reallocation of movement behaviours, we have added the following to the methods on page 14, lines 282-285:  “*For simplicity, the terms adding and subtracting were used to indicate instances of hypothetical reallocations from one movement behaviour to another (e.g., reallocating sleep to MVPA was represented as adding or + 30 minutes of MVPA and subtracting or - 30 minutes of sleep).*” |
| R2.3 | Overall, I think the results need to be written clearer. It is hard to follow what the authors are trying to say, and what are the main messages they want the reader to take away. There are a lot of outcomes and a lot of analyses with the entire 24-hour composition and with the reallocations. All good work, but needs to be clearer. |
|  | As per comment R2.1, the results have been re-written to highlight our findings for each developmental domain, and in doing so we anticipate this section is now clearer. |
| R2.4 | The authors need to consider and mention the implications of the cross-sectional design in the Discussion. |
|  | We have included a sentence pointing out the lack of causal mechanisms in our study on page 28, lines 434-436:  “*A limitation is the cross-sectional study design that prohibits understanding the causal mechanisms of the relationships observed.*” |
| R2.5 (Minor) | Is BMI really a measure of adiposity? I suggest not. Please consider wording around this and changing to something like body size. |
|  | We can appreciate the reviewers point that BMI z-scores are not an ideal measure of adiposity. However, we choose this term to be consistent with the systematic reviews conducted to inform The Canadian 24-Hour Movement Guidelines for the Early Years. To better reflect the limitations of using BMI as a measure of adiposity we have made the following change on page 8, lines 134-135:  “*Heights and weights were measured to calculate the surrogate adiposity measure of body mass index (BMI) z-scores.*” |
| R2.6 | It is not entirely clear where participants were recruited from. This sentence is not clear “Parents or guardians were recruited in Edmonton, Canada and surrounding areas through a local division of Sportball, a program that aims to teach children fundamental sport skills through play, as part of the Parent-Child Movement Behaviours and Pre-School Children’s Development study”. Were they recruited through Sportball, which was part of this other study? Please clarify. |
|  | We have made the following change to make this clearer on pages 5-6, lines 87-92:  “*Data used in this analysis were collected as part of from the Parent-Child Movement Behaviours and Pre-School Children’s Development study. Participants were children aged 3-5 years and their parents, whose primary language at home was English. Parents or guardians were recruited in Edmonton, Canada and surrounding areas through a local division of Sportball, a program that aims to teach children fundamental sport skills through play.*” |
| R2.7 | Line 113: close bracket missing |
|  | We have closed this bracket. |
| R2.8 | How did the sample go from 131 to 95? Why were data missing/invalid? |
|  | We have added the following information on page 15, lines 304-311:  “*From 131 participants, 26 did not receive accelerometers because they were absent from motor development assessments (n=15) or they did not schedule a time for accelerometers to be dropped off after the motor development assessment (n=11). Further, 10 participants did not meet accelerometer wear time criteria. Thus, a total of 95 participants had usable accelerometer data and were included in the analysis. Aside from the analysis of response inhibition (n=93; n=2 software errors) and all motor skills outcomes (n=93, n=2 children chose not to participate), these 95 participants had data for all outcome variables.*” |
| R2.9 | What were the movement behaviour volumes in this cohort compared to the Canadian movement behaviour guidelines for this age group? Would be helpful information for the reader up front in the results. As mentioned in the limitations section. |
|  | We have not added guideline adherence statistics, since this would require the introduction of screen time. However, to make a better comparison in the limitations section, we have added the numerical comparison of our sample to the national average for MVPA. Page 28, lines 439-443:  “*Lastly, convenience sampling from a physical activity program could have limited our generalizability. In fact, the average minutes/day of MVPA in this sample was 40 minutes higher compared to the national average, which could suggest poor generalizability to the broader population of Canadian preschool aged children*”. |
| R2.10 | The higher MVPA of this cohort could be a result of the sampling method. That is, participants were recruited from the Sport Ball program. Please acknowledge this more clearly in methodological considerations. |
|  | We have added the following to the discussion on page 28, lines 439-443:  “*Lastly, convenience sampling from a physical activity program could have limited our generalizability. In fact, the average minutes/day of MVPA in this sample was 40 minutes higher compared to the national average, which could suggest poor generalizability to the broader population of Canadian preschool aged children*”. |
| R2.11 | For Table 1, it would be helpful to see the range of possible scores and the direction (what high/low scores represent) for the outcomes. |
|  | We are hesitant to add more information to this Table, as it is already quite full. However, the reviewer brings up a good point and we have added more context into the methods section to discuss ranges and directions:  Page 8, line 150:  “*Scores for both trials were summed across components to create an object motor skill score and a locomotor skill score, both out of a maximum 48 points.*”  Page 10, lines 191-192  “…*with values closer to 1 indicating better response inhibition.*”  Pages 11-12, lines 222-224:  “*Each subscale ranged from 0 to 5, with values closer to 5 being favourable for sociability, prosocial behaviour, and self-regulation, while values closer to 1 were favourable for internalizing and externalizing.*” |
| R2.12 | Table 1, consider use of decimals. For example, does age really need to be to two decimals (same as in Discussion). |
|  | We have changed the values in Table 1 to one significant digit. |
| R2.13 | Table 2 add a footnote stating that closer to 0=greater codependence. |
|  | We have added the following footnote to Table 2:  “*Values closer to zero indicate higher codependence.*” |
| R2.14 | Table 3, indicate clearly in the title that these were adjusted for covariates. |
|  | The results in Table 3 are not adjusted for covariates. The intention of these first models was to determine if there was an overall relationship with only the movement behaviour composition and the outcome variables. |
