# Supplementary material for: Movement behaviours and physical, cognitive, and social-emotional development in preschool-aged children: Cross-sectional associations using compositional analyses
Source: PLoS One. 2020 Aug 18;15(8):e0237945. doi: 10.1371/journal.pone.0237945 (PMC7433874; doi:10.1371/journal.pone.0237945)
Supplement: Attachment — Submitted filename: Kuzik_P2_PlosOne_Response2 (Aug 3, 2020).docx [file pone.0237945.s002.docx]

| Editor | |
| --- | --- |
| E.1 | I congratulate the authors becase the current study add relevant insights to the scientific literature in this field. Two reviewers completed their reviews in a first round but for the second review only one of them was available. However, I have checked out reviewer's 2 comments and I think there are minor issues still to address (pages and lines are referred to the tracked document): |
|  | We thank the Editor for there review of the manuscript and have addressed their comments below. |
| E.2 | P7 L116-133: Due to the relevance of accelerometry for this study, I think it is important to report the software you used to calculate variables that you used later for analyses. In addition, I do not know why authors selected 30 Hz as sampling frequency when the recommendation for this age group is 90-100 Hz. Also, I understand that 15s epochs are enough, but considering the quick changes in this age group, shorter epochs could provide more confidence to the results. |
|  | We have reported the software used to calculate movement behaviour variables on page 7, line 128:  “*All movement behaviour categorization was conducted in R (version 3.6.1)*”.  We have also added the following to acknowledge the sampling frequency recommended by Migueles et al. (2017) on page 7, lines 115-117:  “*While 90-100 Hz is the recommended frequency for ActiGraph accelerometers in preschool-aged children, we chose 30 Hz to align with the validation studies that our movement behaviour cut-points are based on*”.  Lastly, we have added a similar statement for our selection of epochs on page 7, lines 125-128:  “*While using shorter epochs may be advantageous to better represent the sporadic movement profiles of preschool-aged children, 15-second epochs were used to align with the validation studies that our movement behaviour cut-points are based on*”. |
| E.3 | P14 L287-290: Following reviewer’s 2 recommendations and after reading the sentence you have added respect terms “adding or subtracting”, I encourage to reconsider using terminology that reviewer’s 2 recommend, which I consider more appropriate for that case. |
|  | We have replaced all instances that discussed this procedure in terms of adding or subtracting in favour of reallocations. For instance, on page 23, lines 369-371:  “*Additionally, positive relationships were seen when reallocating LPA with stationary time for locomotor and total motor skills*.” |
| E.4 | P14 L 294: Minutes should be in plural. |
|  | We have changed this instance of minute to minutes on page 15, line 292. |
| E.5 | P15 L310-315: Please, consider to create a flow chart or a figure that could explain easier this flow of participants that you explain at the beginning of the results section. |
|  | We have added a flow chart to the manuscript and changed this portion of the results to reflect this change on page 15, lines 307-311:  “*From 131 participants, a total of 95 participants had usable accelerometer data and were included in the analysis (see Figure 1 for participant flow diagram).*” |
| E.6 | P15 L316: Please, remove a dot after “variables”. It is duplicated. |
|  | We have removed this additional period. |
| E.7 | P18 L330-414: I understand that it is difficult to place tables in the correct point, but I think you have to consider to move tables closer to the text that cites them in order to do it clearer. You cite tables 3, 4 and 5 that are in other parts of the text. Consider that there are a lot of outcomes, all necessaries, but it is important to do it clearer for the reader. |
|  | We have moved Tables 3 and 4 to the paragraph after they are first mentioned. |
